# Prognostic Value of CD166 Expression in Cancers of the Digestive System: A Systematic Review and Meta-Analysis

**DOI:** 10.1371/journal.pone.0070958

**Published:** 2013-08-05

**Authors:** Chao Ni, Zhigang Zhang, Xiaotao Zhu, Yang Liu, Dihong Qu, Ping Wu, Jian Huang, A-xiang Xu

**Affiliations:** 1 Cancer Institute (Key Laboratory of Cancer Prevention and Intervention, National Ministry of Education, Provincial Key Laboratory of Molecular Biology in Medical Sciences), The Second Affiliated Hospital, Zhejiang University School of Medicine, Hangzhou, China; 2 Department of Oncology, Second Affiliated Hospital, Zhejiang University School of Medicine, Hangzhou, China; 3 Department of Urology, the General Hospital of PLA, Beijing, China; Health Canada and University of Ottawa, Canada

## Abstract

**Objective:**

Many studies have reported the prognostic predictive value of CD166 as a cancer stem cell marker in cancers of the digestive system; however, its predictive value remains controversial. Here, we investigate the correlation between CD166 positivity in digestive system cancers and clinicopathological features using meta-analysis.

**Methods:**

A comprehensive search in PubMed and ISI Web of Science through March of 2013 was performed. Only articles containing CD166 antigen immunohistochemical staining in cancers of the digestive system were included,including pancreatic cancer, esophageal cancer, gastric cancer and colorectal cancer. Data comparing 3- and 5-year overall survival along with other clinicopathological features were collected.

**Results:**

Nine studies with 2553 patients who met the inclusion criteria were included for the analysis. The median rate of CD166 immunohistochemical staining expression was 56% (25.4%–76.3%). In colorectal cancer specifically, the results of a fixed-effects model indicated that CD166-positive expression was an independent marker associated with a smaller tumor burden (T category; RR = 0.93, 95%, CI: 0.88–0.98) but worse spread to nearby lymph nodes (N category; RR = 1.17, 95% CI: 1.05–1.30). The 5-year overall survival rate was showed relationship with cytoplasmic positive staining of CD166 (RR = 1.47 95% 1.21–1.79), but no significant association was found in the pool or any other stratified analysis with 3- or 5- year overall survival rate.

**Conclusion:**

Based on the published studies, different cellular location of CD166 has distinct prognostic value and cytoplasmic positive expression is associated with worse prognosis outcome. Besides, our results also find CD166 expression indicate advanced T category and N-positive status in colorectal cancer specifically.

## Introduction

Although treatments for digestive system cancers have been developing rapidly, these cancers, especially pancreatic and colorectal cancer, are still responsible for a number of deaths [1]. It has been reported that a small subpopulation of cells, called cancer stem cells (CSCs), dominate the initiation, progression, relapse and metastasis of cancer. In recent years, certain cell surface markers have been reported as CSC markers in digestive system cancers, with high expression of these markers usually an indicator of poor prognosis. Among them, CD166 has been identified experimentally as a putative stem cell marker in various cancers [2]–[5] with a high capacity for sphere and xenograft formation.

CD166, also known as activated leukocyte cell adhesion molecule (ALCAM), is a highly conserved 110-kDa multidomain transmembrane type-1 glycoprotein of the immunoglobulin super family, which was first described as a CD6 ligand on leukocytes [6]. Further studies have revealed that it is broadly expressed in different tissues and cells, including neuronal, immune and epithelial cells, as well as stem cells of hematopoietic and mesenchymal origin. CD166 plays an important role in many biological activities, including T-cell activation and proliferation, angiogenesis, hematopoiesis and axon fasciculation [7]. CD166 is also closely related to various cancers, including melanoma, prostate cancer and breast cancer. Many cancers of the digestive system have also been found to have high expression of CD166; however, the prognostic outcomes of these studies are contradictory. Two earlier studies by Weichert and Horst et al [8], [9] reported positive expression of CD166 in colorectal cancer and that CD166 was an independent prognostic marker associated with poor survival rates. However, two other recent independent studies have found conflicting outcomes [10], [11].

Because the contradiction may be caused by limited sample sizes along with other factors, here, we performed a pooled analysis of all cancers of the digestive system, including pancreatic, gastric, esophageal and colorectal cancer. Although all cancer types were derived from the digestive system, heterogeneity within the different tissues may also exist. Therefore, we analyzed pancreatic and colorectal cancer independently to improve accuracy. Here, we present a meta-analysis that aims to clarify the prognostic value of CD166 in digestive system cancers based on currently published evidence. Other clinicopathological features were also examined in this study.

## Materials and Methods

### Search Strategy

We performed a systematic literature search of the electronic databases PubMed and ISI Web of Science up to March of 2013. Search terms included “CD166 antigen”, “ALCAM” or “activated leukocyte cell adhesion molecule” with “cancer”, “neoplasm” or “carcinoma”. The titles and abstracts of potential references were carefully examined to exclude irrelevant studies; the remaining articles within the topic of interest were reviewed in depth for their relevance.

### Selection Criteria

The studies in this meta-analysis included either randomized control studies (RCTs) or observational studies (case-control or cohort) that evaluated the relationship between CD166 expression and the risk of developing a digestive system cancer. Studies were included if they met the following criteria: (a) they focused on digestive system cancers (esophageal, gastric, pancreatic and colorectal cancer); (b) they defined a CD166-positive group by immunohistochemistry; and (c) they described a correlation between CD166, clinicopathological features and survival outcome (either disease free survival or overall survival). Articles were excluded from the analyses based on the following criteria: (a) non-English papers; (b) review articles or letters; and, (c) insufficient data to determine the RR and CI, or the full text could not be found.

Based on a critical review checklist provided by the Dutch Cochrane Centre [12] and in an effort to control the quality of this meta-analysis, we examined the quality of all the included studies. Seven key points are depicted here: (a) clear definition of study population and origin of country, (b) clear definition of the type of carcinoma, (c) clear definition of the study design, (d) clear definition of the outcome assessment, (e) clear definition of the cut-off of CD166 expression, (f) clear definition of the method of CD166 assessment and (g) sufficient time of follow-up.

### Data Extraction

All data were extracted by two independent reviewers. Data tables were generated to extract all relevant data from texts, tables and figures, including: author, year, country, patient number, detection method, duration of follow-up, T category, N category, distant metastasis, positive rates of CD166 overexpression, as well as overall survival (OS) rate. For articles that only provided survival data in a Kaplan-Meier curve, the software GetData Graph Digitizer 2.24 (http://getdata-graph-digitizer.com/) was applied to digitize and extract the data.

Because the cut-off score for CD166 positivity varied among the studies, we defined the CD166 positive group with respect to the original articles. Because of the high degree of malignancy and poor outcome of pancreatic cancer patients in clinic, the OS was standardized to 3-years in pancreatic cancer, and the other cancer types were standardized to 5-years.

### Statistical Analysis

The statistical analysis was performed according to the guidelines proposed by the Meta-Analysis of Observational Studies in Epidemiology group. Relative risk (RR) with 95% confidence interval (95% CI) was calculated with Review Manager 4.2. The heterogeneity among studies was measured using the Q and I^2^ tests. A Fixed or Random model was used depending on the heterogeneity analysis. The potential for publication bias was assessed using the Begg rank correlation method and the Egger weighted regression method (software stata11.0). P value <0.05 was considered statistically significant. All P values are two-tailed.

## Results

### Search Results

**Figure 1:**
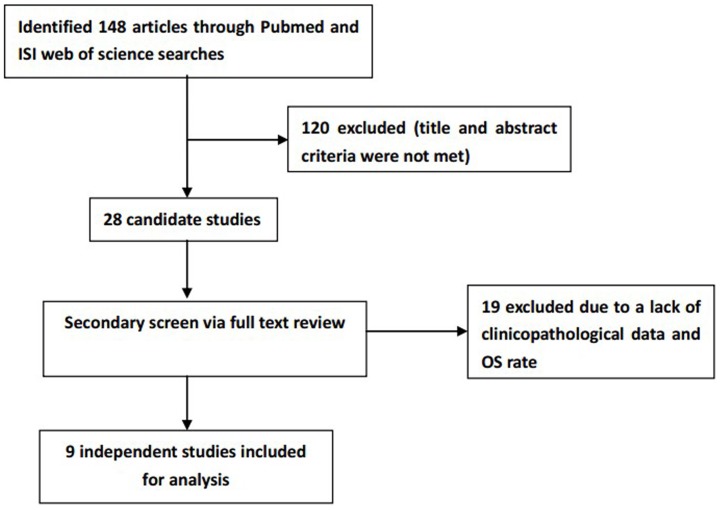
Flow chart for the selection of articles to include.

Initially, 148 articles were retrieved utilizing the search strategy above. From the title and abstract review, 120 of the articles were excluded due to non-human experiments, non-digestive system cancer-related studies, or non-original articles (e.g., review, letter). Of the remaining articles, 19 were excluded because they did not provide clinicopathological data, particularly the OS rate [2]–[4], [13]–[28]. Finally, a total of 9 studies were included in the meta-analysis with 2553 participants. All of these studies explicitly assessed the expression of CD166 and risk of cancer death by immunohistochemical staining (Figure 1).

### Study Characteristics

All features of the 9 eligible studies are listed in Table 1. Among them, 7 were from Germany, 1 from Japan and 1 from Switzerland. Furthermore, 2 studies focused on pancreatic cancer, 4 focused on colorectal cancer, and 1 focused on each of pancreatic neuroendocrine, esophageal and gastric cancer. A total of 2553 patients with a median of 188 (from 38 to 1274) per study were included. The TNM stage and tumor grade was reported in 8 and 7 studies, respectively. Furthermore, 6 studies determined CD166 expression only on the membrane, and 3 studies stained CD166 on both the membrane and within the cytoplasm. Tissue microarrays for CD166 expression analysis were utilized in 5 studies. Three studies used whole tissue immunochemistry staining and 1 study applied both methods (Table 1). In 6 studies, none of the patients received neo-adjuvant radio- or chemotherapy prior to surgery [8]–[10], [29]–[31]. Pertaining directly to colorectal cancer, patients with tumor recurrence in Horst’s study were treated with chemotherapy, radiation therapy or surgical resection when possible. In Lugli’s study, 478 patients received post-operative therapy. In Kahlert’s study, 88 cases had an R0 resection, and 9 cases had an R1 resection. For gastric cancer, all patients had an R0 resection with at least a D1 lymph node dissection.

**Table 1:** Characteristics of included studies.

NO.	Author	Year	Country	Number of Patients	Organ	Duration of Follow-up	Methods	Staining patterns	Cut off scores (pos/neg)	T category (T1–2/3–4)	N category (P/N)	Distant metastasis(M0/M1)	Grade (1–2/3)	OS (5 years)
**1**	Tachezy	2012	germany	192	pancretic CA	0–193 months (median 14 months)	TMA	membrane staining	membrane staining positive(72/120)	H(105/15); L(65/7)	NA	NA	H(68/52); L(38/34)	H(29/91); L(6/66)*
**2**	Tachezy	2011	germany	38	pancretic neuroendocrine CA	7–168 months (median 45 months)	TMA	membrane staining	2+ >30% and 3+ (28/10)	NA	NA	H(4/23);L(5/5)	H(26/2); L(9/1)	H (23/51);L (3/7)*
**3**	Kahlert	2009	germany	97	pancretic CA	median 18.5 months	whole tissue	membrane and cytoplasmic staining	membrane and cytoplasmic staining (30/67)	H(6/24); L(12/55)	H(6/24); L(12/55)	NA	H(15/15); L(46/21)	H (0/30) L(19/48)*
**4**	Tachezy	2012	germany	289	esophageal CA	0–178 months (median 16 months)	TMA	membrane staining	1+ >30% or≥2+ (204/85)	H(87/117); L(35/50)	H(75/129); L(26/59)	H(166/38); L(72/13)	H(128/76); L(39/46)	H (43/163) L(30/55)*
**5**	Ishigami	2011	japan	142	gastric cancer	0.3–104.5 months (median 18.6 months)	whole tissue	membrane and cytoplasmic staining	membrane and cytoplasmic staining (36/106)	H(28/8); L(79/27)	H(23/13); L(41/65)	NA	NA	H (20/16) L (71/35)*
**6**	weichert	2004	germany	111	colorectal CA	median 47 months	whole tissue	membrane and cytoplasmic staining	membrane and cytoplasmic staining (34/77)	NA	NA	NA	NA	H (20/14) L (61/16)*
**7**	Horst	2009	germany	110	colorectal CA	4.8–162 months (median 94.8 months)	TMA	membrane staining	membrane staining positive (70/40)	H(22/48); L(17/28)	H(12/54); L(7/33)	NA	H(63/7); L(36/4)	H (42/28) L (33/7)*
**8**	Lugli	2010	switzerland	1274	colorectal CA	0–80 months	TMA and whole tissue	membrane staining	membrane staining positive (775/499)	H(165/594); L(72/417)	H(417/329); L(228/253)	H(275/63); L(70/18)	H(658/100); L(438/49)	H (465/310) L (274/225)
**9**	Tachezy	2012	germany	300	colorectal CA	1–180 months (median 39 months)	TMA	membrane staining	membrane staining positive (229/71)	H(59/170); L(14/57)	H(115/114); L(31/40)	H(168/61); L(53/18)	H(200/29); L(50/21)	H (135/94); L(26/45)*

CA: cancer; H: high expression; L: low expression; NA:not available; TMA: tissue microarray.

* data read by GetData Graph Digitizer.

### Correlation of CD166 to Clinical Features

The correlation of CD166 membrane expression with overall T category, N category, distant metastasis and tumor grade is illustrated in Figures S1, S2, S3 and S4. The results suggest that CD166 correlated more with T1 and T2 category patients (pooled RR = 0.94, 95% CI: 0.89–0.99) and N-positive patients (RR = 1.20, 95% CI: 1.09–1.32). However, in colorectal cancer specifically, CD166 expression was associated with more advanced T category (RR = 0.93, 95% CI: 0.88–0.98) and N-positive status (RR = 1.17, 95%CI 1.05–1.30), and it did not show any relationship with other kinds of digestive tumors. Furthermore, we stratified the extracted data by geographic area, staining pattern, follow-up time and sample size: studies in Europe showed CD166 expression was related with T1 and T2 category patients (RR = 0.94, 95% CI: 0.89–0.99; membrane staining: RR = 0.94, 95% CI: 0.89–0.99) and N-positive status (RR = 1.17, 95% CI: 1.06–1.30; membrane staining: RR = 0.94, 95% CI: 0.89–0.99). Membranous staining of CD166 also revealed related with T1 and T2 category patients (RR = 0.94, 95% CI: 0.89–0.99), and both staining patterns were showed associated with N-positive status (membrane staining: RR = 0.94, 95% CI: 0.89–0.99; membrane & cytoplasmic staining RR = 1.51, 95% CI: 1.09–2.10). Studies with shorter (RR = 1.34, 95% CI: 1.04–1.71) or longer follow-up times (RR = 1.17, 95% CI: 1.05–1.30) both showed a positive relationship between CD166 expression and N status. The same result was also found in both studies with smaller (RR = 1.40, 95% CI: 1.02–1.92) or larger sample sizes (RR = 1.18, 95% CI: 1.06–1.30). However, there was no clear association between CD166 expression and other clinicopathological features including distant metastasis (RR = 1.10, 95% CI: 0.85–1.43) or tumor grade (RR = 0.90, 95% CI: 0.63–1.27) in either the overall or stratified analyses (Table 2).

**Table 2:** Results of meta-analysis on CD166 expression.

		T category(T3,4 vs. T1,2)		N category(positive vs. negative)		Distant metastasis(M1 vs. M0)		Grade(grade 3 vs. grade 1,2)		OS 3year(death vs. survive)		OS 5year(death vs. survive)
	N1	RR(95%CI)	N2	RR(95%CI)	N3	RR(95%CI)	N4	RR(95%CI)	N5	RR(95%CI)	N6	RR(95%CI)
Over all	7	0.94(0.89–0.99)	6	1.20(1.09–1.32)	4	1.10(0.85–1.43)	7	0.90(0.63–1.27)	2	0.90(0.80–1.02)	9	1.11(0.88–1.04)
Cancer type^a^												
PC	2	1.04(0.81–1.34)	1	1.12(0.46–2.69)	1	1.70(0.90–3.23)	3	1.07(0.82–1.39)	2	0.90(0.80–1.02)	3	1.04(0.70–1.55)
EC	1	0.98(0.79–1.21)	1	1.20(0.83–1.74)	1	1.22(0.68–2.17)	1	0.69(0.53–0.90)	0	NA	1	1.22(1.03–1.45)
GC	1	0.87(0.44–1.74)	1	1.65(1.17–2.33)	0	NA	0	NA	0	NA	1	1.35(0.85–2.12)
CRC	3	0.93(0.88–0.98)	3	1.17(1.05–1.30)	2	0.98(0.71–1.36)	3	0.81(0.35–1.92)	0	NA	4	1.18(0.64–2.16)
Geographic area												
Europe	6	0.94(0.89–0.99)	5	1.17(1.06–1.30)	4	1.10(0.85–1.43)	7	0.90(0.63–1.27)	2	0.90(0.80–1.02)	8	1.09(0.85–1.39)
Asia	1	0.87(0.44–1.74)	1	1.65(1.17–2.33)	0	NA	0	NA	0	NA	1	1.35(0.85–2.12)
Staining pattern												
Membrane	5	0.94(0.89–0.99)	4	1.17(1.06–1.30)	4	1.10(0.85–1.43)	6	0.81(0.56–1.15)	1	0.95(0.80–1.02)	6	0.96(0.74–1.24)
Membrane &cytoplasmic	2	0.95(0.74–1.21)	2	1.51(1.09–2.10)	0	NA	1	1.60(0.96–2.64)	1	0.83(0.70–0.98)	3	1.47(1.21–1.79)
Follow time (month)^b^												
<37.5	4	0.99(0.84–1.16)	3	1.34(1.04–1.71)	1	1.22(0.68–2.17)	3	0.96(0.63–1.45)	2	0.90(0.80–1.02)	4	1.15(0.86–1.54)
≥37.5	3	0.93(0.88–0.99)	3	1.17(1.05–1.30)	3	1.06(0.79–1.42)	4	0.81(0.37–1.75)	0	NA	5	1.11(0.71–1.76)
Sample size^c^												
<188	3	1.01(0.84–1.21)	3	1.40(1.02–1.92)	1	1.70(0.90–3.23)	3	1.37(0.87–2.18)	1	0.83(0.70–0.98)	5	1.49(1.24–1.79)
≥188	4	0.93(0.88–0.98)		1.18(1.06–1.30)	3	1.04(0.78–1.38)	4	0.79(0.53–1.19)	1	0.95(0.80–1.12)	4	0.87(0.66–1.15)

a PC:pancretic cancer, EC: esophageal cancer, GC: gastric cancer, CRC: colorectal cancer.

b median of followup time among all studies included.

c median of sample size among all studies included.

### CD166 Expression and 3- or 5-year Overall Survival Rate

**Figure 2:**
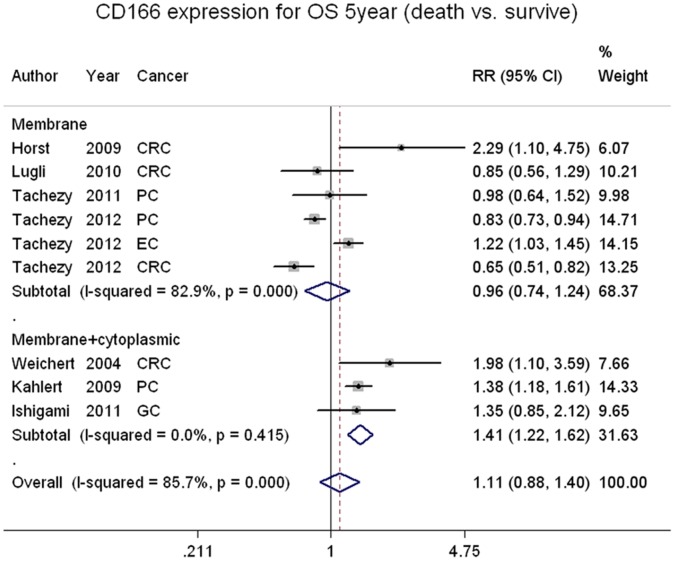
CD166 stratified on staining pattern and 5-year overall survival rate in digestive cancer patients.

The 5-year overall survival rate was extracted from 9 studies, which was composed of 4 colorectal cancer studies, 1 gastric, 1 esophageal and 3 pancreatic cancer study and two studies of pancreatic cancer were also examined with 3-year overall survival. The pooled 5-year overall survival rates of CD166-positive and CD166-negative patients were 57.3% (767/1339) and 33.7% (523/1214), respectively. The 3-year overall survival rates were 25.3% (38/150) and 12.2% (17/139) for the same patients, respectively. The pool analysis did not show significant association between overall survival rate and CD166 status (Figures S5 and S6), however, the stratified group based on staining pattern revealed membrane and cytoplasmic staining was related with worse prognosis (RR = 1.47 95%CI 1.21–1.79), but the membrane staining alone could not show any prediction value (Table 2, Figure 2).

### Publication Bias

Heterogeneity testing and publication bias analyses were performed among the studies based on membrane and cytoplasmic staining. Results indicate that the funnel plots are almost symmetric and that the P values of Begg’s and Egger’s tests were 0.296 and 0.533, respectively (Table S1). Thus, no evidence for publication bias in the meta-analysis was found.

## Discussion

Many carcinomas of the digestive system have been considered cancer stem cell-related diseases in recent years, including esophageal, gastric, pancreatic and colorectal cancer [32]–[35]. In an effort to identify these small populations of cells, a number of cell surface proteins have been identified as CSC markers. Many studies have demonstrated experimentally that CD166 can enrich for CSC-like cells in a variety of cancers [4], [5]. Moreover, Levin et al [4] have found that CD166 is expressed at low levels in differentiated intestinal cells but robustly expressed on the surface of cells within the stem cell niche at the base of a crypt, which strongly infers its relationship with stem cell properties. Although high expression of CSC markers are usually considered as a prognosis of poor outcome, several contradictions to this generalization exist in published studies on the putative CSC-marker CD166 [21].

To investigate the basis for these contradictory conclusions, the staining methods among the studies in this meta-analysis were compared. Although the same antibody was used in each of these studies, the staining methods have varied between analyzing both membranous and cytoplasmic staining, whereas others take only membranous staining in consideration. Tachezy et al [11] found that CD166 was predominantly expressed at the cell membrane and that cytoplasmic staining intensity was related to the intensity of membranous staining and did not occur in the absence of membranous staining; thus, only membranous staining was considered in their study. The other three studies in this meta-analysis that focused on membranous staining including colorectal and pancreatic cancer, all concluded that high CD166 expression is a positive marker for good prognosis [10], [14], [36]. In contrast, the three studies in our analysis that analyze both membrane and cytoplasmic staining intensity propose that CD166 high expression contributes to poor clinical outcome [8], [30], [31]. Interestingly, in oral, breast and ovarian carcinomas, decreased membranous and increased cytoplasmic expression of CD166 is also associated with worse prognosis [37]–[39].

CD166 has been demonstrated to participate in the metastatic cascade of cancer cells. CD166-mediated intercellular adhesion involves interactions between the amino terminal D1 domain of opposing receptor molecules on two cells and is strengthened by lateral oligomerization of neighboring molecules on the cell surface, which engage the membrane proximal domains D4 and D5 [40]. These data suggest that high CD166 expression could impede cancer cell release from a local lesion. Furthermore, it has been shown that CD166/ALCAM can be actively cleaved by ADAM17/TACE-mediated proteolysis [41]. In ovarian cancer, pharmacologic inhibition of ADAM proteins, or specific silencing of ADAM17/TACE, hampered shedding of CD166 expressed on the cell surface. Interestingly, CD166/ALCAM can be translocated from the cell surface to the cytoplasm via a clathrin-dependent pathway. Specifically, soluble CD166/ALCAM (sALCAM) binds to scFv I/F8 to form a chimera, which induces endocytosis of the membrane-bound CD166/ALCAM. Recombinant sALCAM chimeric molecules inhibit the adhesive function of CD166/ALCAM through a competitive binding effect, which results in increased cancer cell motility [42], [43]. Van Kempen et al [44] also found that disruption of CD166 self-interaction was associated with tumor cell motility and metastasis. These studies all suggest that CD166 shedding from the cell surface may predict tumor progression and poor prognosis.

Colon cancer is a classical model for tumor progression studies because of its natural development from crypt stem cells to adenomas to fully formed carcinomas [45]; CD166 is highly expressed on the surface of crypt cells in this disease. However, both cell surface and cytoplasmic expression of CD166 is apparent in early adenoma formation in Apc^Min/+^ mice, human colorectal cancer and metastatic disease. Furthermore, only a subset of CD166 positive cells co-localize with the proliferation marker Ki67 [4]. These observations suggest that specific subcellular localization of CD166 could be used as a clinical prognostic marker because the loss of CD166 cell surface expression appears to be a precursor for tumor progression.

Part of the shedding CD166 would release into the tumor environment and circulation. A few studies have gone so far as to examine the shedding of CD166/ALCAM into blood serum. Two studies in this meta-analysis of esophageal and pancreatic cancer found a significant upregulation of CD166 in the blood serum of patients, but this observation only had a prognostic value in the esophageal cancer patients, as no significant correlation was found between elevated tissue expression and serum level in the pancreatic cancer patients [29], [36]. Klasingam et al. [46] have described significantly high sALCAM in the blood serum of breast cancer patients, but no prognostic data were reported. Variation in sALCAM among studies may have multiple causes: sALCAM must pass through the barrier of tumor tissue and vascular endothelial cells to be flushed into the blood stream, and sequential sectioning has failed to establish a direct relationship between ADAM17/TACE and ALCAM [31]. These data imply that the level of sALCAM in circulation is unstable and that it is inappropriate to use it for estimating prognoses.

Although the evidence addressed above may imply that cell surface expression of CD166 would be a positive prognostic marker and that the shedding of CD166, in other words, cytoplasmic CD166 expression would predict the reverse outcome, but in our stratified analysis, only cytoplasmic staining showed close relationship with poor prognosis, and the result of membrane staining of CD166 in unclear because two of the included studies provided significantly contradictory results [9], [29]. Recently, a colorectal cancer study may have provided a succinct method to assess the prognostic value of CD166 [21]. They found that an elevated mRNA level of CD166 was associated with poor outcome, yet intact membranous CD166 protein (co-localized extracellular and intracellular domain) is associated with improved outcome. With a novel method that stained the extracellular and intracellular domains of CD166 separately, they found that the extracellular domain of CD166 underwent shedding while the intracellular epitope remained. Thus, they concluded that shedding of the extracellular domain of CD166 correlated with patient outcome rather than loss of expression, which was previously considered the prognostic value of CD166. Unfortunately, the antibody applied in previous studies could not differentiate the subcellular epitope of CD166 in immunohistochemistry, making it difficult for a pathologist to accurately judge whether the protein was located on the cell surface, greatly affecting the prognostic capacity of CD166 expression.

This meta-analysis is subject to a few limitations. First, the number of studies included is relatively small, particularly for gastric and esophageal cancer. Second, 8 of the 9 studies were from Europe, including 7 from Germany, 1 from Switzerland and only one from Asia. Distinct site differences are believed to exist and could cause publication bias. Third, the criteria for determining positive or negative expression of CD166 varied among studies. Six studies only studied membranous CD166 expression while the rest also took cytoplasmic expression under consideration. Finally, although we tried to identify the disease free survival rate, these data were almost entirely missing from these studies. Most importantly, based on our meta-analysis of previous studies and systematic review of related articles indicates that the biological function of CD166 in tumor progression is complicated and that determining its subcellular location could be the key for accurate prognostic predictions. Thus, a more standardized staining method should be employed in future studies.

## Supporting Information

Figure S1 **CD166 expression and T category.**
(TIF)

Figure S2 **CD166 expression and N category.**
(TIF)

Figure S3 **CD166 expression and tumor grade.**
(TIF)

Figure S4 **CD166 expression and distant metastasis.**
(TIF)

Figure S5 **CD166 expression and 5-year overall survival rate.**
(TIF)

Figure S6 **CD166 expression and 3-year overall survival rate.**
(TIF)

Table S1 **Heterogeneity test and publication bias analyses among studies included.**
(DOC)

File S1 **PRISMA Flow Diagram.**
(DOC)

File S2 **PRISMA Checklist.**
(DOC)
