# Supplementary figures and images for: Prognostic Value of CD166 Expression in Cancers of the Digestive System: A Systematic Review and Meta-Analysis
Source: PLoS One. 2013 Aug 5;8(8):e70958. doi: 10.1371/journal.pone.0070958 (PMC3733726; doi:10.1371/journal.pone.0070958)

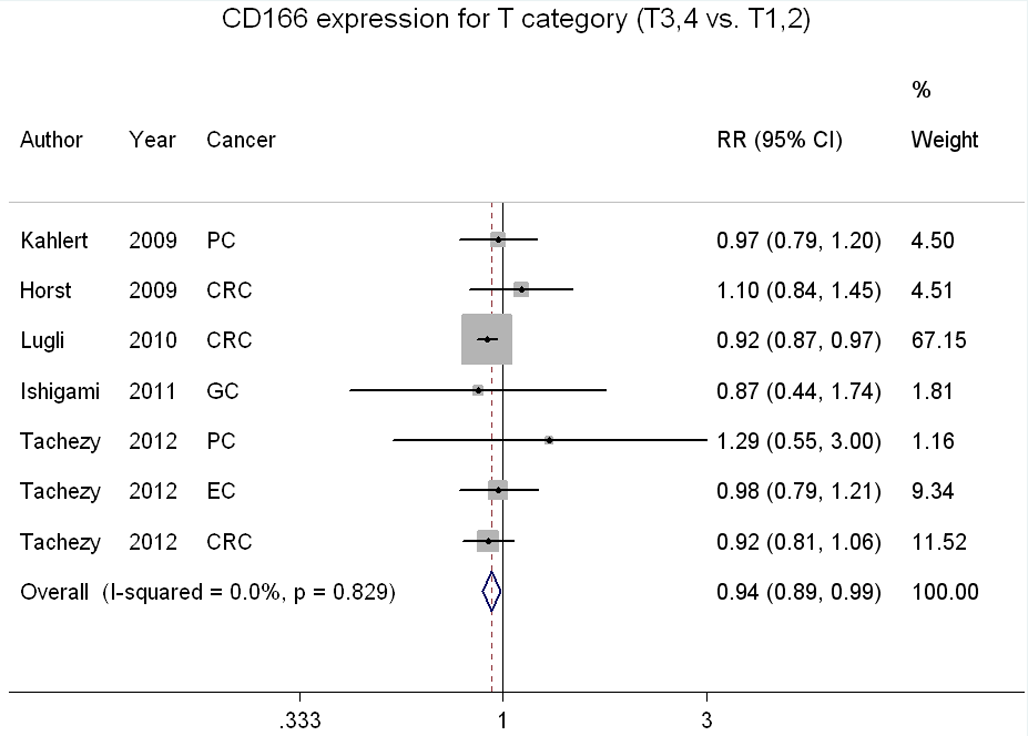

Supplement: Figure S1 — CD166 expression and T category. (TIF) [file pone.0070958.s001.tif]

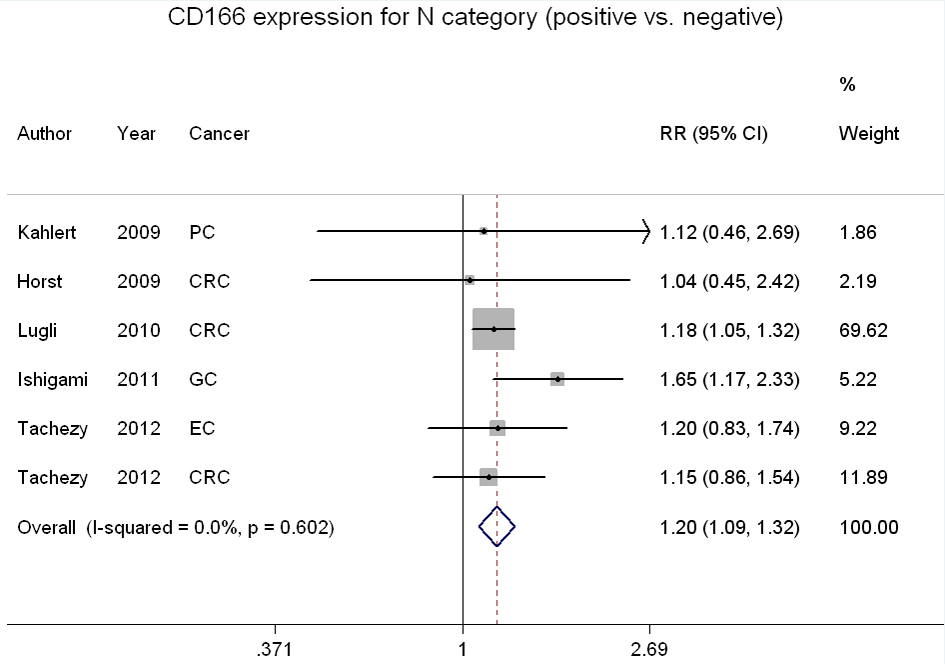

Supplement: Figure S2 — CD166 expression and N category. (TIF) [file pone.0070958.s002.tif]

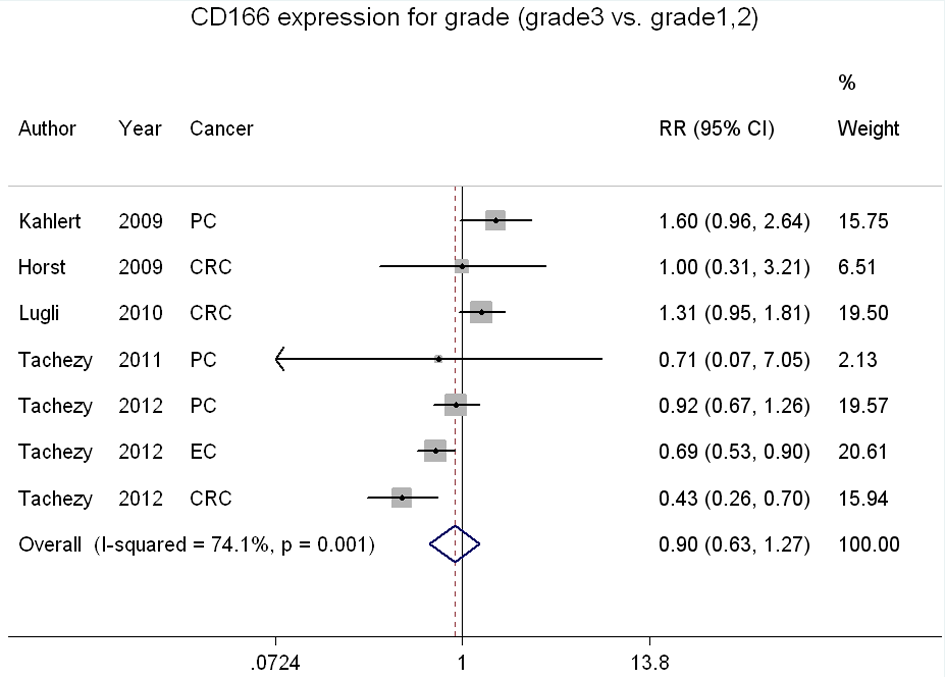

Supplement: Figure S3 — CD166 expression and tumor grade. (TIF) [file pone.0070958.s003.tif]

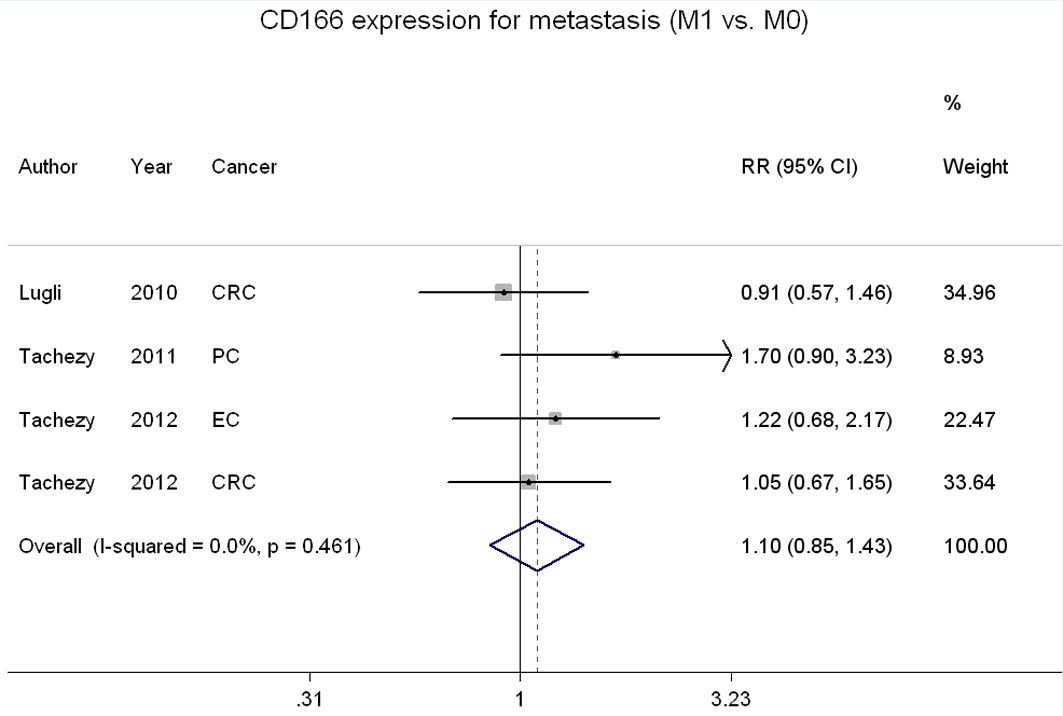

Supplement: Figure S4 — CD166 expression and distant metastasis. (TIF) [file pone.0070958.s004.tif]

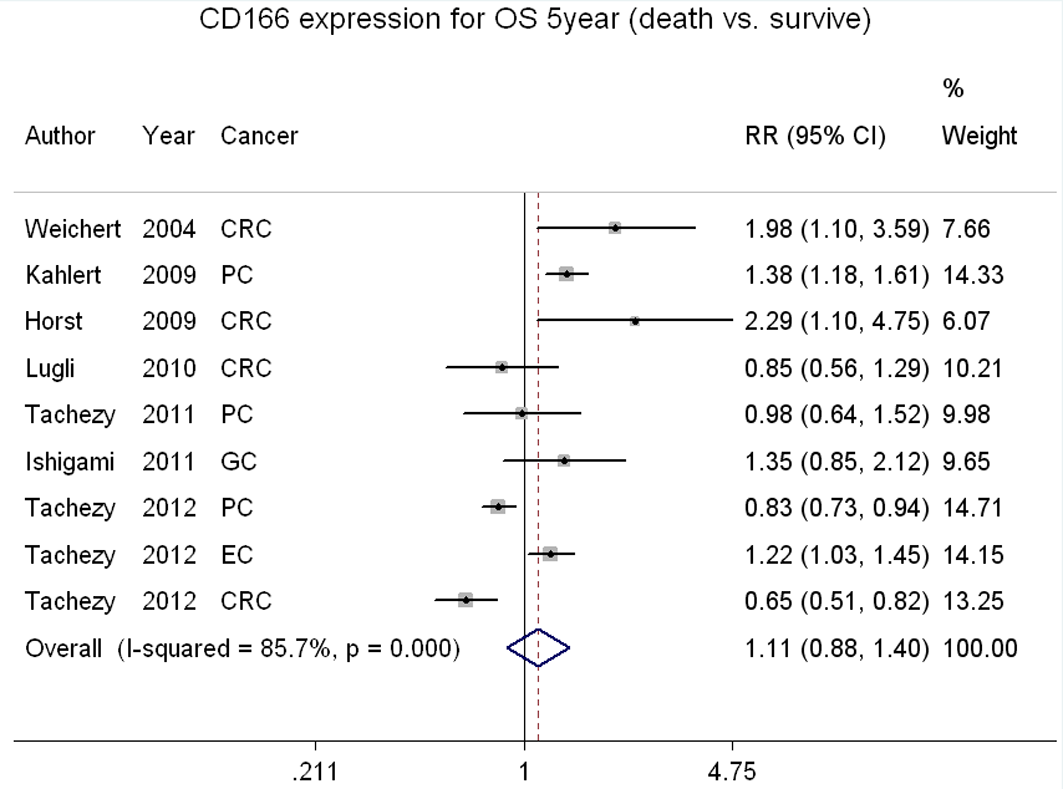

Supplement: Figure S5 — CD166 expression and 5-year overall survival rate. (TIF) [file pone.0070958.s005.tif]

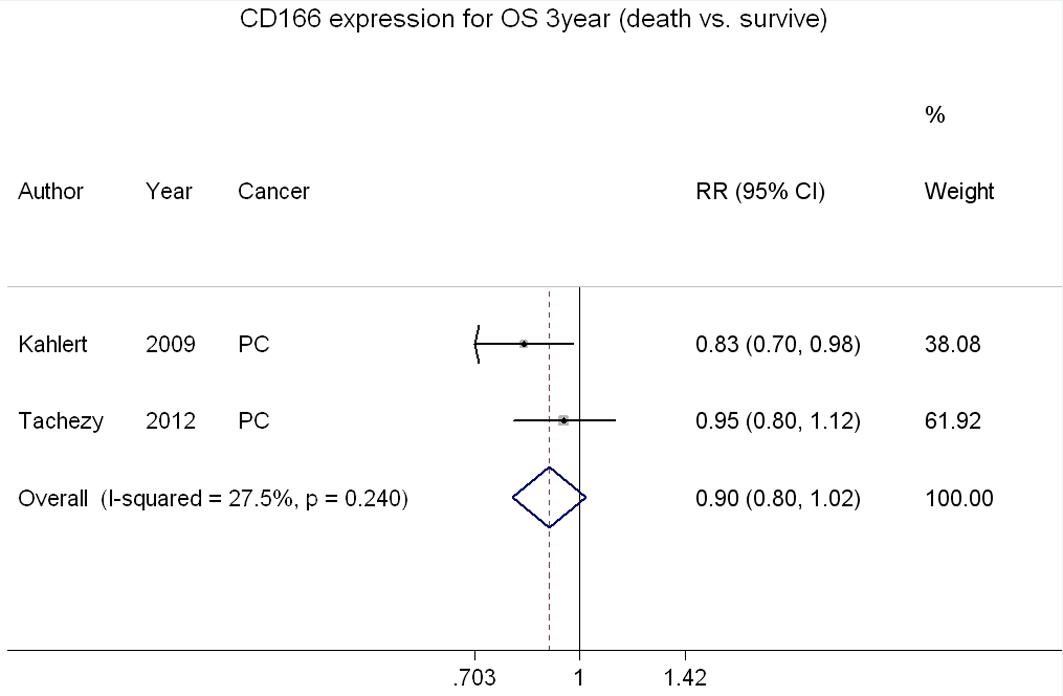

Supplement: Figure S6 — CD166 expression and 3-year overall survival rate. (TIF) [file pone.0070958.s006.tif]
