# Supplementary material for: Prognostic Value of CD166 Expression in Cancers of the Digestive System: A Systematic Review and Meta-Analysis
Source: PLoS One. 2013 Aug 5;8(8):e70958. doi: 10.1371/journal.pone.0070958 (PMC3733726; doi:10.1371/journal.pone.0070958)
Supplement: Table S1 — Heterogeneity test and publication bias analyses among studies included. (DOC) [file pone.0070958.s007.doc]

| **Table S1.** Heterogeneity test and publication bias analyses among studies included | | | | | | | | | | | | | | | | | | | | | | | |
| --- | --- | --- | --- | --- | --- | --- | --- | --- | --- | --- | --- | --- | --- | --- | --- | --- | --- | --- | --- | --- | --- | --- | --- |
|  | T category (T3,4 vs. T1,2) | | | | |  | N category (positive vs. negative) | | | | |  | Distant metastasis (M1 vs. M0) | | | | |  | Grade (grade 3 vs. grade 1,2) | | | | |
|  | Heterogeneity | |  | Publication bias | |  | Heterogeneity | |  | Publication bias | |  | Heterogeneity | |  | Publication bias | |  | Heterogeneity | |  | Publication bias | |
|  | *P* ^a^ | *I*²(%) ^b^ |  | *P* ^c^ | *P* ^d^ |  | *P* ^a^ | *I*²(%) ^b^ |  | *P* ^c^ | *P* ^d^ |  | *P* ^a^ | *I*²(%) ^b^ |  | *P* ^c^ | *P* ^d^ |  | *P* ^a^ | *I*²(%) ^b^ |  | *P* ^c^ | *P* ^d^ |
| Over all | 0.829 | 0 |  | 0.230 | 0.066 |  | 0.602 | 0 |  | 0.707 | 0.719 |  | 0.461 | 0 |  | 0.308 | 0.093 |  | 0.001 | 74.1 |  | 1.000 | 0.936 |
| Cancer type |  |  |  |  |  |  |  |  |  |  |  |  |  |  |  |  |  |  |  |  |  |  |  |
| PC | 0.443 | 0 |  | 1.000 | - |  | - | 0 |  | - | - |  | - | 0 |  | - | - |  | 0.180 | 41.7 |  | 1.000 | 0.893 |
| EC | - | 0 |  | - | - |  | - | 0 |  | - | - |  | - | 0 |  | - | - |  | - | 0 |  | - | - |
| GC | - | 0 |  | - | - |  | - | 0 |  | - | - |  | - | - |  | - | - |  | - | - |  | - | - |
| AVA/AVC | - | - |  | - | - |  | - | - |  | - | - |  | - | - |  | - | - |  | - | - |  | - | - |
| CRC | 0.436 | 0 |  | 0.296 | 0.365 |  | 0.949 | 0 |  | 0.296 | 0.065 |  | 0.668 | 0 |  | 1.00 | - |  | 0.001 | 85.8 |  | 1.000 | 0.753 |
| Geographic area |  |  |  |  |  |  |  |  |  |  |  |  |  |  |  |  |  |  |  |  |  |  |  |
| Europe | 0.725 | 0 |  | 0.06 | 0.017 |  | 0.998 | 0 |  | 0.462 | 0.217 |  | 0.461 | 0 |  | 0.308 | 0.093 |  | 0.001 | 74.1 |  | 1.000 | 0.936 |
| Asia | - | 0 |  | - | - |  | - | 0 |  | - | - |  | - | - |  | - | - |  | - | - |  | - | - |
| Staining pattern |  |  |  |  |  |  |  |  |  |  |  |  |  |  |  |  |  |  |  |  |  |  |  |
| Membrane | 0.619 | 0 |  | 0.086 | 0.047 |  | 0.989 | 0 |  | 0.308 | 0.370 |  | 0.461 | 0 |  | 0.308 | 0.093 |  | 0.004 | 71.5 |  | 1.000 | 0.845 |
| Membrane/cytoplasmic | 0.716 | 0 |  | 1.000 | - |  | 0.399 | 0 |  | 1.000 | - |  | - | - |  | - | - |  | - | 0 |  | - | - |
| Follow time (month) |  |  |  |  |  |  |  |  |  |  |  |  |  |  |  |  |  |  |  |  |  |  |  |
| <37.5 | 0.915 | 0 |  | 0.734 | 0.643 |  | 0.378 | 0 |  | 1.000 | 0.694 |  | - | 0 |  | - | - |  | 0.013 | 76.9 |  | 0.296 | 0.105 |
| ≥37.5 | 0.436 | 0 |  | 0.296 | 0.365 |  | 0.949 | 0 |  | 0.296 | 0.065 |  | 0.285 | 20.3 |  | 1.000 | 0.235 |  | 0.003 | 78.7 |  | 0.734 | 0.702 |
| Sample size |  |  |  |  |  |  |  |  |  |  |  |  |  |  |  |  |  |  |  |  |  |  |  |
| <188 | 0.718 | 0 |  | 1.000 | 0.831 |  | 0.444 | 0 |  | 1.000 | 0.086 |  | - | 0 |  | - | - |  | 0.626 | 0 |  | 1.000 | 0.153 |
| ≥188 | 0.805 | 0 |  | 0.089 | 0.066 |  | 0.982 | 0 |  | 1.000 | 0.920 |  | 0.743 | 0 |  | 1.000 | 0.458 |  | 0.001 | 82.8 |  | 1.000 | 0.633 |
|  | | | | | | | | | | | | | | | | | | | | | | | |

| **Table S1 (continued)** | | | | | | | | | | | | |
| --- | --- | --- | --- | --- | --- | --- | --- | --- | --- | --- | --- | --- |
|  | OS 3year | | | | |  | OS 5year | | | | | |
|  | Heterogeneity | |  | Publication bias | |  | Heterogeneity | |  | Publication bias | | |
|  | *P* ^a^ | *I*²(%) ^b^ |  | *P* ^c^ | *P* ^d^ |  | *P* ^a^ | *I*²(%) ^b^ |  | *P* ^c^ | *P* ^d^ | |
| Over all | 0.240 | 27.5 |  | 1.000 | - |  | <0.001 | 85.7 |  | 0.466 | 0.466 | |
| Cancer type |  |  |  |  |  |  |  |  |  |  |  | |
| PC | 0.240 | 27.5 |  | 1.000 | - |  | <0.001 | 92.2 |  | 1.000 | 0.850 | |
| EC | - | - |  | - | - |  |  | 0 |  | - | - | |
| GC | - | - |  | - | - |  | - | 0 |  | - | - | |
| AVA/AVC | - | - |  | - | - |  | - | - |  | - | - | |
| CRC | - | - |  | - | - |  | 0 | 86.1 |  | 0.308 | 0.032 | |
| Geographic area |  |  |  |  |  |  |  |  |  |  |  | |
| Europe | 0.240 | 27.5 |  | 1.000 | - |  | <0.001 | 87.1 |  | 0.711 | 0.548 | |
| Asia | - | - |  | - | - |  | 0 | - |  | - | - | |
| Staining pattern |  |  |  |  |  |  |  |  |  |  |  | |
| Membrane | - | 0 |  | - | - |  | <0.001 | 82.9 |  | 0.452 | 0.599 | |
| Membrane/cytoplasmic | - | 0 |  | - | - |  | 0.451 | 0 |  | 0.296 | 0.533 | |
| Follow time (month) |  |  |  |  |  |  |  |  |  |  |  | |
| <37.5 | 0.240 | 27.5 |  | 1.000 | - |  | <0.001 | 90.3 |  | 1.000 | 0.510 | |
| ≥37.5 | - | - |  | - | - |  | 0 | 81.6 |  | 0.086 | 0.007 | |
| Sample size |  |  |  |  |  |  |  |  |  |  |  | |
| <188 | - | 0 |  | - | - |  | 0.149 | 40.8 |  | 0.221 | 0.615 | |
| ≥188 | - | 0 |  | - | - |  | <0.001 | 86.1 |  | 1.000 | 0.894 | |
| ^a^ *P* for Q test.  ^b^ Proportion of between-study heterogeneity accounting for total heterogeneity.  ^c^ *P* values of Begg’s test.  ^d^ *P* values of Egger’s test. | | | | | | | | | | | |  |
