# Supplementary material for: Prognostic Value of CD166 Expression in Cancers of the Digestive System: A Systematic Review and Meta-Analysis
Source: PLoS One. 2013 Aug 5;8(8):e70958. doi: 10.1371/journal.pone.0070958 (PMC3733726; doi:10.1371/journal.pone.0070958)
Supplement: File S1 — PRISMA Flow Diagram. (DOC) [file pone.0070958.s008.doc]

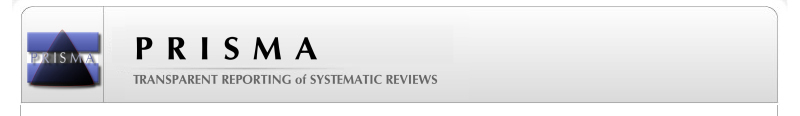
**PRISMA 2009 Flow Diagram**

**Screening**

**Included**

**Eligibility**

**Identification**

Records identified through pubmed and ISI web of knowledge database searching
(n =203 )

28 articles retrieved for detailed assessment

Totally 175 Records excluded

138 irrelavant

11 review

24 no desirable outcome

2 patients without appropriate control

Articles excluded, with reasons:

without clinicopathological and overall survival data

n=19

9 articles included in final analysis
